# Prostate malignant tumor and benign prostatic hyperplasia microenvironments in black African men: Limited infiltration of CD8+ T lymphocytes, NK‐cells, and high frequency of CD73+ stromal cells

**DOI:** 10.1002/cnr2.1817

**Published:** 2023-04-24

**Authors:** Pélagie Mougola Bissiengou, Jérôme Gaston Montcho Comlan, Gabrielle Atsame Ebang, Maguette Sylla Niang, Joel Fleury Djoba Siawaya

**Affiliations:** ^1^ Service d'Immunologie, Département des Sciences Fondamentales, Faculté de Médecine Université des Sciences de la Santé Libreville Gabon; ^2^ Service d'Immunologie, Département des Sciences Biologiques et Pharmaceutiques Appliquées, Faculté de Médecine, de Pharmacie et d'Odontostomatologie Université Cheikh Anta Diop Dakar Senegal; ^3^ Unité d'anatomie‐Cytologie‐Pathologie Centre Hospitalier Universitaire de Libreville Libreville Gabon; ^4^ Service Laboratoire Centre Hospitalier Universitaire Mère‐Enfant Fondation Jeanne EBORI Libreville Gabon

**Keywords:** benign prostatic hyperplasia, cancer, CD73, CD8 T‐lymphocytes, natural killer, prostate

## Abstract

**Background:**

Anti‐cancerous immunology has yet to be investigated in the African black population, despite being the dawn of precision medicine.

**Aim:**

Here we investigated the tumor microenvironment of prostate cancer and benign prostatic hyperplasia (BPH) in black Africans.

**Methods:**

Through immunohistochemistry analysis of prostate cancer and BPH patients' biopsies, we investigated the expression and distribution of CD73, CCD8 T‐lymphocytes, and natural killer cells. In addition, we looked at tumor‐infiltrating features CD8 T‐lymphocytes and natural killer cells.

**Results:**

We show for the first time in black Africans a high expression of CD73 in epithelial‐stromal cells and virtually no infiltration of CD8 T lymphocytes and natural killer cells in the tumoral area. In addition, CD73 was seven (7) times more likely to be expressed in prostate cancer stromal tissues than in benign prostatic hyperplasia tissues (odds ratio = 7.2; χ^2^ = 21; *p* < .0001). In addition, PSA concentration was significantly higher in prostate cancer patients than in BPH patients (*p* < .001). Also, the PSA‐based ROC. analysis showed an area under the curve of 0.87 (*p* < .0001).

**Conclusion:**

CD73 expression is more likely expressed in prostate cancer stromal tissues than in benign prostatic hyperplasia tissues. The features of prostate cancer in Black Africans suggest CD73 expression as a possible target for immunotherapy in this population.

## 1 INTRODUCTION

Prostate cancer is an uncontrolled malignant growth of mutated cells from the prostate (a gland of the male reproductive system). The cancerous cells can metastasize to other parts of the body. The majority of prostate cancers are adenocarcinomas (90%). Benign prostatic hyperplasia (BPH), for which lower urinary tract symptoms are common manifestations, affects more than half of men above 65 years old.^1^ Prostate cancer occurs independently of benign prostatic hyperplasia (BPH).

With around 1.4 million new cases and 375 000 deaths worldwide, prostate cancer was the most common cancer and the fifth leading cause of death among men over 60 in 2020.^2^ In Africa, with an incidence of 29.7 cases per 100 000 people and a mortality rate of 16.3 cases per 100 000, prostate cancer is becoming a public concern.^2^ The high mortality rate in this region is mainly attributed to the stage at which the disease is diagnosed. 75% of diagnoses are advanced, and the disease is immediately aggressive, particularly in those under 60 years of age.^3^, ^4^ In Gabon, the incidence of prostate cancer was estimated to be between 30 and 42 newly diagnosed cases per 100 000 inhabitants. The mortality was estimated between 14 and 19 cases per 100 000 inhabitants.^4^

Benign prostatic hyperplasia (BPH), a pathology for which lower urinary tract symptoms are common manifestations, affects more than half of men above 65 years old.^1^ Although Prostate cancer occurs independently of benign prostatic hyperplasia, there might be some overlapping in symptoms, biomarker expression, or secretion.^5^ Prostate cancers are most often detected based on elevated blood levels of prostate‐specific antigen, a glycoprotein (PSA > 4 ng/mL) generally expressed by prostate tissue,^3^ and nonspecific lower urinary tract symptoms.^6^ Also, common prostate diseases, including BPH, and prostatitis, have varying degrees of elevated blood PSA levels and nonspecific lower urinary tract symptoms (LUTS).^5^, ^6^ Therefore, a tissue biopsy is a gold standard for confirming prostate cancer and defining the tumor microenvironment.

The tumor microenvironment (TME) is a complex heterogeneous milieu built around different cell types (immune cells, tumor cells, and stromal cells) and a network of molecules produced by these cells. This complex microenvironment dictates tumor differentiation, dissemination, immune evasion, and even resistance to therapy.^7^

The standard therapeutic strategy against prostate cancer is based on palliative treatment based on androgen deprivation, aimed at reducing testosterone levels.^8^ However, castration resistance becomes inevitable after an average period of 18 months, typically requiring the introduction of systemic chemotherapy with docetaxel.^9^ Castration‐resistant forms now benefit from new hormone therapies (abiraterone acetate and enzalutamide), new systemic chemotherapy (cabazitaxel), and bone‐tropic targeted therapies against bone metastases such as denosumab.^10^, ^11^, ^12^ These treatments can be combined with complementary radiotherapy and/or chemotherapy, depending on case.^13^ Nevertheless, the ability of the tumor to develop resistance to anti‐androgenic therapies often compromises the prognosis of patients. Less than a third of them survive 5 years after diagnosis.^14^

The development of new therapeutic approaches to circumvent the progression of these tumors represents an increasingly crucial and urgent need for managing this type of cancer. Among the many concepts under study, augmenting the immune response to cancer has been proposed as a valid therapeutic option, offering an alternative approach to improving survival.^15^, ^16^ For some years, the idea that immunotherapy could be the solution has become more evident. Evasion of the immune system has recently been recognized as one of the defining properties of a cancer cell.^17^, ^18^

The close interactions between tumor epithelial cells and the microenvironment, which are dynamic over time, establish immunosuppression. This is a defining feature of cancer and a crucial step in tumor progression and establishing metastases.^7^, ^19^, ^20^ Several immunosuppression mechanisms can be put in place by the tumor microenvironment to decrease the antitumor efficacy of the immune system.^20^ Adenosine generated by the membrane enzyme CD73 is one of these factors.^21^ The involvement of this enzyme in alterations in the regulation of major pathophysiological processes, including carcinogenesis and immune escape from tumors, including prostate cancer, has been reported.^21^, ^22^, ^23^, ^24^

Within this context, we explored the immune microenvironment of prostate cancer in African men. We mainly looked at CD73 expression within the tumor and stromal zone and the tumor‐infiltrating capability of CD8 T‐lymphocytes and natural killer cells.

## 2 MATERIALS AND METHODS

This is a cross‐sectional cytological, histological, and Immunohistochemical (IHC)^25^ analysis of biopsies from prostate cancer and BPH patients.

### 2.1 Patients

The target population was men aged 40 and above who had a prostate biopsy and were diagnosed with prostate cancer or BPH between January 2018 and December 2020 by the Pathological Anatomy and Cytology Laboratory of the Faculty of Medicine in Libreville.

Patients were selected based on their medical records and histopathology analysis results. We included patients with well‐defined cancer histopathology analysis results and enough biopsy samples to perform immunohistochemistry. Patients with undefined histopathological results and insufficient biopsies for immunohistochemistry analysis were excluded.

The medical and histopathology records included anthropometric, clinical, bloodwork (including PSA), and social data. Also recorded were the organ removed, the sample type, the sample preanalytical treatment, the number of cores obtained during the biopsy, the biopsy approach, and the final diagnosis.

For each patient, we retrieved information about their age, the concentration of total prostate‐specific antigen (ng/mL), the number of cores obtained during the biopsy, and the Gleason score. Patients with less than 12 cores at biopsy, follow‐up biopsies after diagnosis, transurethral resection of the prostate, and an unspecified prostate‐specific antigen value, were not included.

The diagnosis of prostate cancer and BPH was established according to architectural and cytological criteria established by the International Society of Urological Pathology in 2014.^26^ At this point, 134 patients were eligible for the study. Patients were further categorized as indicated in the study flowchart (Flowchart 1).

**FLOWCHART 1:**
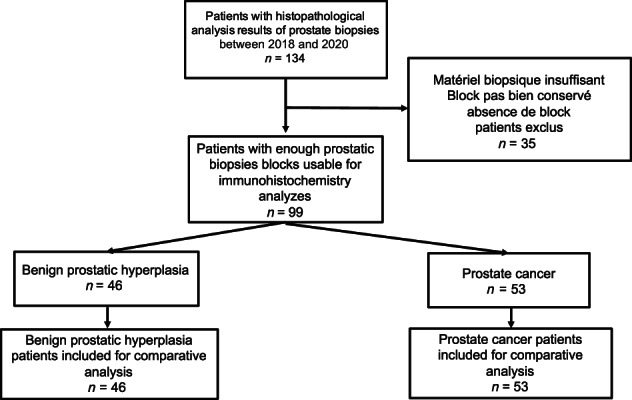
Study flowchart.

### 2.2 Collection and morphological analysis

Stored prostate biopsy samples fixed with 10% buffered formalin and embedded in paraffin were first comparatively analyzed and matched to the Hematoxylin‐Eosinstained slide used for the initial diagnosis. Based on this analysis, samples from 100 out of 134 patients were eligible for an Immunohistochemical study (53 prostate cancers and 47 BPH patients). The 34 remaining patients were excluded due to missing or low‐integrity samples.

### 2.3 Oversee independent analysis of biopsy samples

All the selected blocks of biopsy samples were sent to France at the “France Tissue Bank” laboratory in Fréjus for a second independent analysis and immunohistochemistry‐based analysis. Two (2) pathologists from the “France Tissue Bank” laboratory (France) confirmed prostate cancer and benign prostatic hypertrophy diagnosis made in Libreville (Gabon).

### 2.4 Immunohistochemistry

All immunohistochemistry tests were done by France Tissue Bank” laboratory (France: https://www.francetissuebank.com) in collaboration with the MEDIPATH Group (France: https://medipath.fr). Immunohistochemical analysis was performed on fresh white 3‐micrometer slides obtained using the Microtome micron HM 325 (Dako Agilent Technologies, France). Deparaffinization, unmasking, and staining procedures were performed on the DAKO Coverstainer automated system (Dako Agilent Technologies, France). The Leica Bond III instrument (Leica Biosystems Newcastle) and its standard reagents were used in the immunohistochemistry of CD8 T lymphocytes. The Benchmark Ultra instrument and its standard reagents (VENTANA/Roche Tucson) were used for immunohistochemistry marking CD73 positive cells and CD56 Natural Killer cells. All the antibodies used were monoclonal to be more specific in detecting the proteins sought (CD8, CD56, CD73). All was done according to each manufacturer's instructions and procedures. The data on antibodies used (source/catalog number) are shown in Tables 1 and S1.

**TABLE 1:** Source, catalog number, and characteristics of reagent used.

Antibody	Antibody clone	Manufacturer reference	Supplier	Antigen unmasking	Dilution	Incubation time	Detection kit
CD8	4811	NCL‐L‐CD8‐4B11	Leica Biosystèms	pH 9	1:50	20 min	Bond Polymer
CD56	MRQ‐42	760–4596	Roche	pH 8, 4	Prediluted	16 min	Ultra view
CD73	RM431	BSB‐3716‐3	BioSB	pH 8, 4	Prediluted	20 min	Ultra view

#### 2.4.1 CD73 and CD56 labeling

Fresh slides with tissue Sections 3 micrometers in diameter were dried on a hot plate at 50°C for 20 min. The slides were then loaded onto the BenchMark® instrument together with antibodies (monoclonal rabbit anti‐CD73/NT5E IgG (clone RM431 from Bio S.B.) or monoclonal rabbit anti‐CD56 IgG1 from Cell Marque—Sigma Aldrich) diluted at 1:50. Also loaded was the ultraViewTM detection kit (from VENTANA/Roche Tucson).

Pretreatment with light CC1 was chosen to deparaffinize and unmask antigens. The unmasking of the antigen was carried out in a buffer solution with a pH of 8.4. Antibodies were incubated for 15 min at 37°C before starting the staining cycle. Once the staining cycle was complete, the slides were removed from the instrument, washed in the Tween 20 (2% in phosphate buffered saline [PBS]) washing buffer (Thermo Fisher Scientific), and then rinsed with plenty of water. Next, slides were plunged in a 96% benzyl alcohol bath for 3 minutes and then in a xylene bath for 1 and 3 min. Finally, slides were mounted on the SAKURA tissue‐tek film E2 slide mounter (Sakura Finetek).

#### 2.4.2 CD8 labeling

The fresh slides obtained after sections of three micrometers in diameter of the samples are dried on a hot plate at 50°C. for 20 min. The slides are coated with covers tile and then loaded onto the Leica Bond III instrument, along with the ready‐to‐use monoclonal mouse anti‐CD8 antibody (IgG2b clone 4811, Leica Biosystems Newcastle) and the detection kit. The slide was incubated at 60°C for 30 min. After this incubation, deparaffinization was done at a temperature of 72°C for 30 s, followed by hydration in alcohol baths (70°, 80°, and 90°). Antigen unmasking was performed by heating samples at 100°C for 20 min in BOND ER solution (pH 9) 2 (Leica Biosystems Newcastle). Endogenous peroxidase was blocked by incubating sections with 0.3% hydrogen peroxide for 5 min. Next, sections were coated with primary antibodies, and slides were incubated for 15 min at 37°C. The slides were then washed with buffered saline, followed by 10 min incubation with a post‐primary block at room temperature. After three washes with deionized water, slides were counterstained with hematoxylin (Dako Agilent Technologies) for 5 min. Finish by dipping the slides in a 96% benzyl alcohol bath for 3 min and then in a xylene bath for between 1 and 3 min.

#### 2.4.3 Controls

We used amygdala tissues to control the reading and interpretation of CD8+ T cell labeling. The latter has the characteristic of expressing CD8 T lymphocytes in the normal state. For the reading and interpretation of CD56+ marked cells, we chose the neuroblastoma tissue as a positive control because neuroblastoma strongly expresses CD56. Placental tissue served as a positive control for reading CD73 expression as recommended by the manufacturer. Negative control slides without the primary antibody were included in all lots.

### 2.5 Reading and digitalization of stained slides

Two pathologists independently scored positive slides. CD73+, CD56+, and CD8+ were counted in five randomly selected high‐power fields at ×40 magnification, and counts were scored positive or negative. Each slide was digitized using a Philips UFS ×40 magnification scanner (Philips) in TIFF format. Complementary information on the material used can be found in Data S1.

### 2.6 Statistical analysis

The statistical analysis was performed using GraphPad Prism software version 6. A chi‐square test, a measure of the difference in frequencies of the outcomes of a set of events or variables (observed frequencies in one or more categories), was used to compare the rate at which the targeted markers occurred in different groups of participants or histological sections. The odds ratio (OR) that quantifies the association's strength between two events was calculated using the contingency table. Thus, OR was used to quantify the association's strength between cancer and its microenvironment cell phenotypes. The Mann–Whitney *U* test was performed to compare the concentrations of PSA in BPH patients vs. prostate cancer patients.

Additionally, the receiver operating characteristic (ROC) Curve analysis was used to establish the ability of a PSA to discriminate prostate cancer cases from BPH cases. The threshold of significance was set at a p‐value below 0.05.

### 2.7 Ethics and administrative authorization

Ethical approval was obtained from the institutional ethics committee. All data in this study were de‐identified, and the ethics committee waived informed consent. Export authorization for human samples for scientific research under the French Public Health Code Articles R1235‐7 and R1235‐8 was obtained.

## 3 RESULTS

### 3.1 Characteristics of prostate cancer patients

**FIGURE 1:**
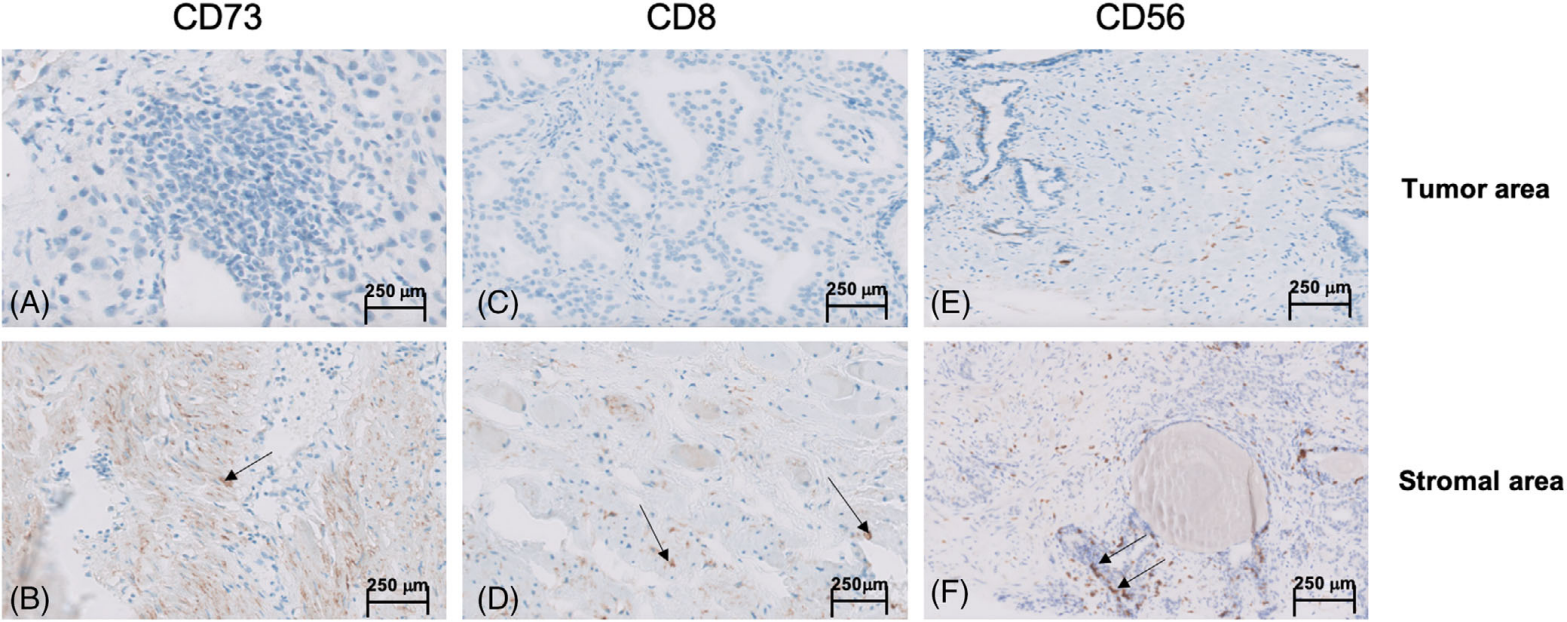
Marked CD73+ cells and CD8+ T‐lymphocytes infiltration as well as natural killer cells (NK‐cells) determined by immunohistochemistry: (A, C and E) Cells expressing CD73, CD8, and CD56 in the tumor area, (B, D, and F) Cells expressing CD73, CD8, and CD56 in the stromal (×40).

Nearly 72% of cancer patients had a perineural invasion of cancerous cells. An overview of CD73+ cells, CD8+ T‐Lymphocytes, and NK‐Cells (CD56+) distribution in cancer patients' tumoral and stromal tissues is represented in Figure 1A–F.

#### 3.1.1 CD73 distribution in prostate cancer patients, tumoral versus stromal area

**FIGURE 2:**
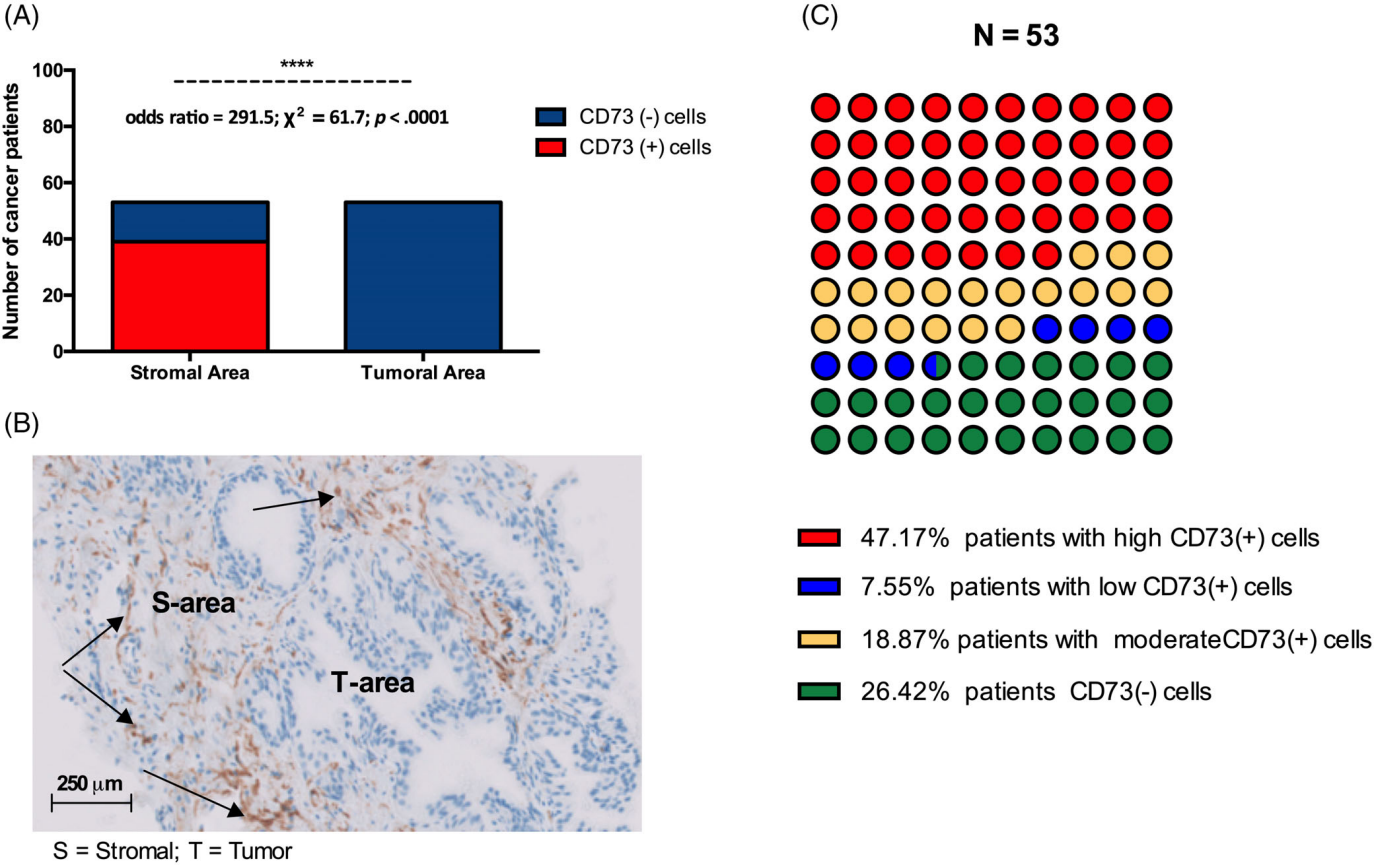
CD73 expression in the tumor zone and the stromal zone. (A) Cells expressing CD73 were exclusively located in the stromal area of cancer patients. (B) Illustration of CD73+ cells distribution in the stromal and tumor areas (the brown staining illustrates the presence of CD73+ cells). (C) 74% of cancer patients expressed CD73).

CD73 was utterly absent from patients' tumoral area, whereas nearly 74% (39/53) of patients CD73 marked cells in their stromal area (odds ratio = 291.5; χ^2^ = 61.7; *p* < .0001) (Figure 2A,B). Stromal CD73+ cells were high in 47.1%, moderate in 19%, low in 7.5%, and absent in 26.4% of prostate cancer patients (Figure 2C).

#### 3.1.2 CD8+ T‐Lymphocytes distribution in prostate cancer patients, tumoral versus stromal area

**FIGURE 3:**
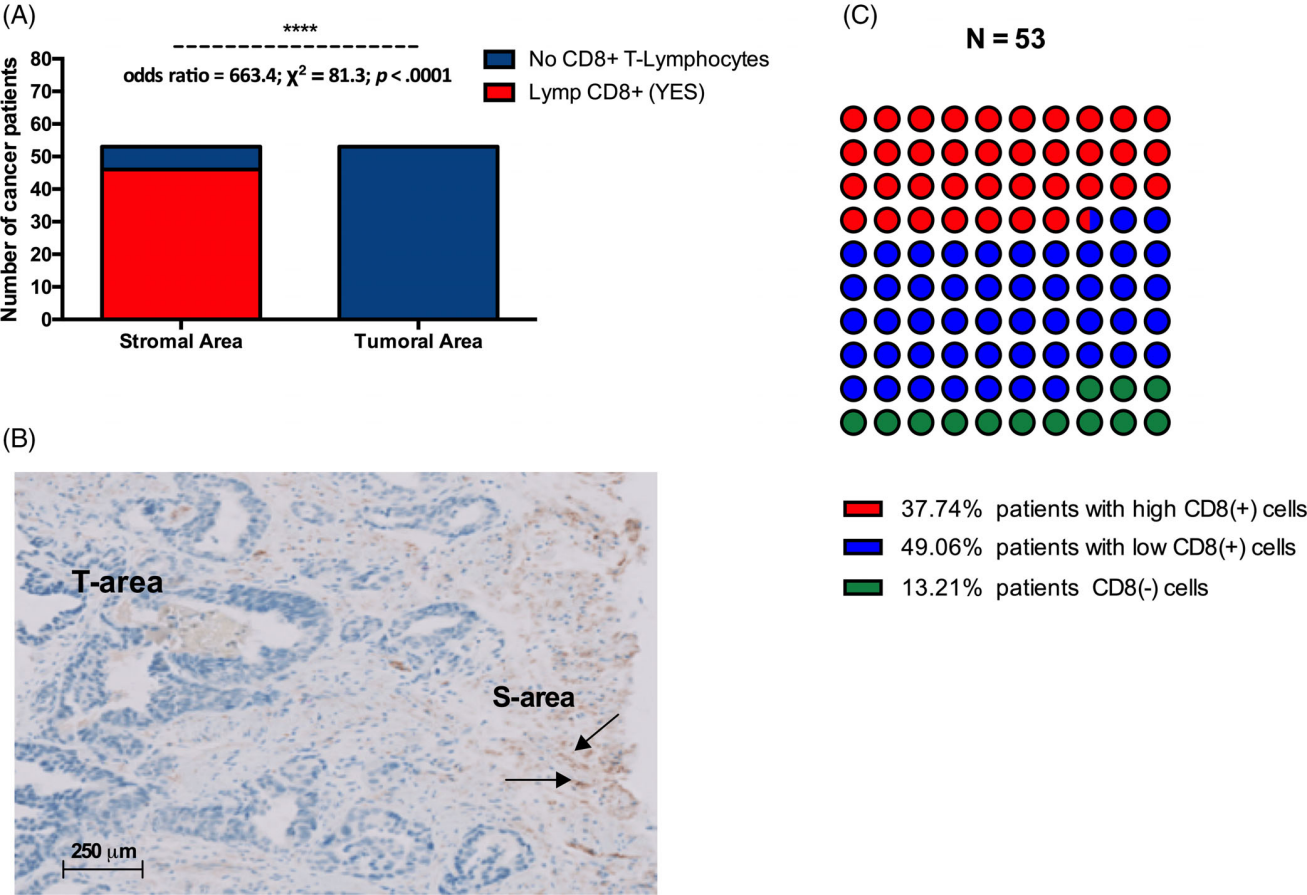
Distribution of CD8+ T lymphocytes in the stromal zone and the tumor zone. (A) CD8+ T‐lymphocytes were exclusively located in the stromal area of cancer patients. (B) Illustration of CD8+ T lymphocytes distribution in the stromal and tumor areas (the brown staining illustrates the infiltration of CD8+ T lymphocytes). (C) 87% of cancer patients had CD8+ T‐lymphocytes in the stromal area).

No Lymphocytes T CD8+ infiltration was observed in the tumoral area of patients. 87% of (46/53) patients had CD8+ T‐Lymphocytes present in their stromal area (odds ratio = 663.4; χ^2^ = 81.3; *p* < .0001) (Figure 3A,B). Looking at the stromal concentration of CD8+ T‐Lymphocytes, 38% of patients had a high stromal concentration of CD8+ T‐Lymphocytes, and 49% had a low number of stromal CD8+ T‐Lymphocytes and 13% had no CD8+ T‐Lymphocytes in their stromal area (Figure 3C).

#### 3.1.3 NK cells (CD56+) distribution in prostate cancer patients, tumoral versus stromal area

**FIGURE 4:**
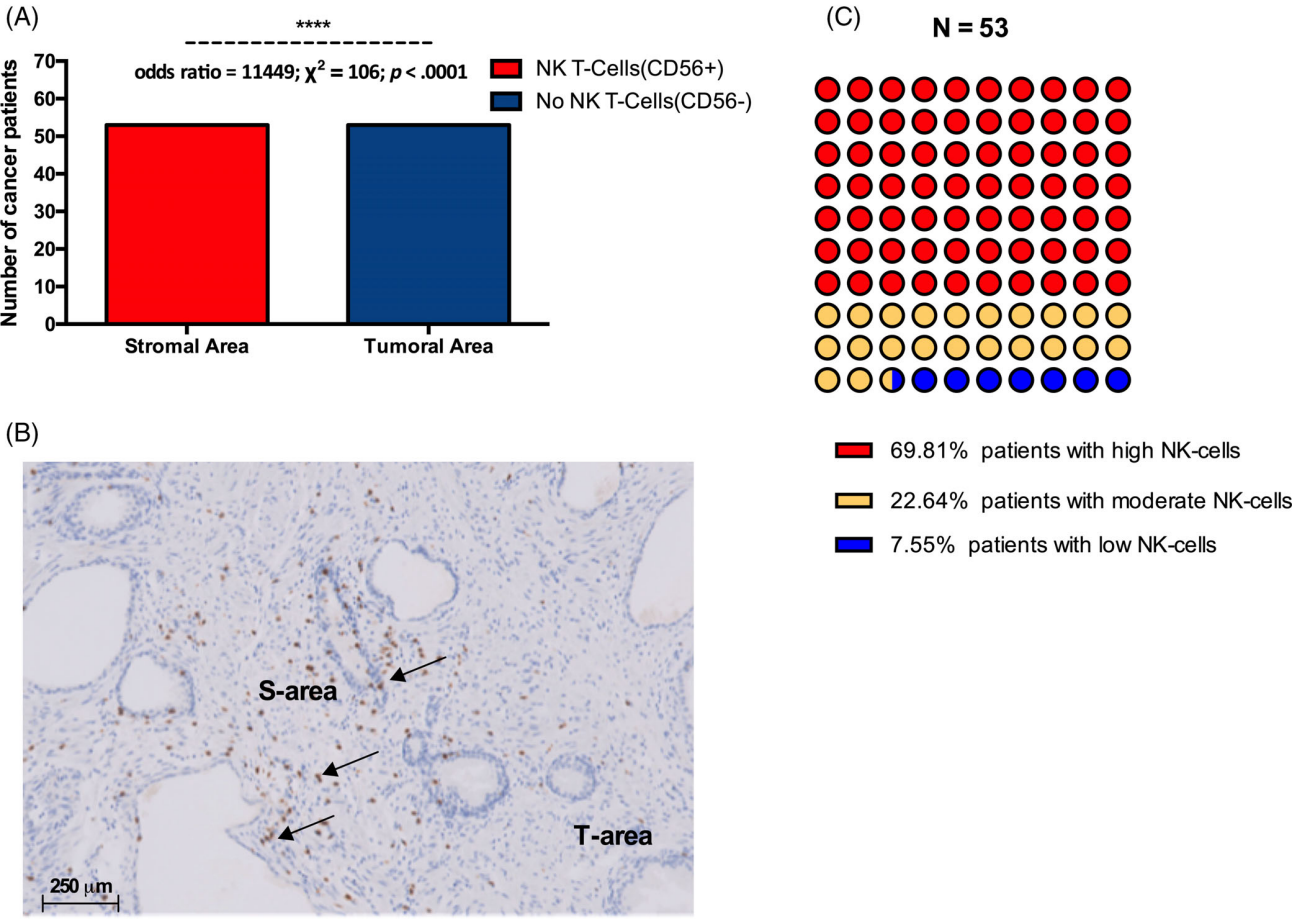
Distribution of NK cells (CD56+ cells) are distributed in the stromal and tumor zones. (A) NK cells were exclusively located in the stromal area of cancer patients. (B) Illustration of NK cells distribution in the stromal and tumor areas (the brown staining illustrates NK‐cells' infiltration). (C) All cancer patients had NK cells in the stromal area).

All cancer patients had NK cells (CD56+ cells) in their stromal area, but no NK cells infiltrated the tumoral area (odds ratio = 11 449; χ^2^ = 106; *p* < .0001) (Figure 4A,B). Stromal NK cells were high in 69.8%, moderate in 22.6%, and low in 7.5% of prostate cancer patients (Figure 4C).

### 3.2 Comparison between prostate cancer versus BPH patients

#### 3.2.1 Stromal CD73+ cells distribution, cancer versus BPH patients

**FIGURE 5:**
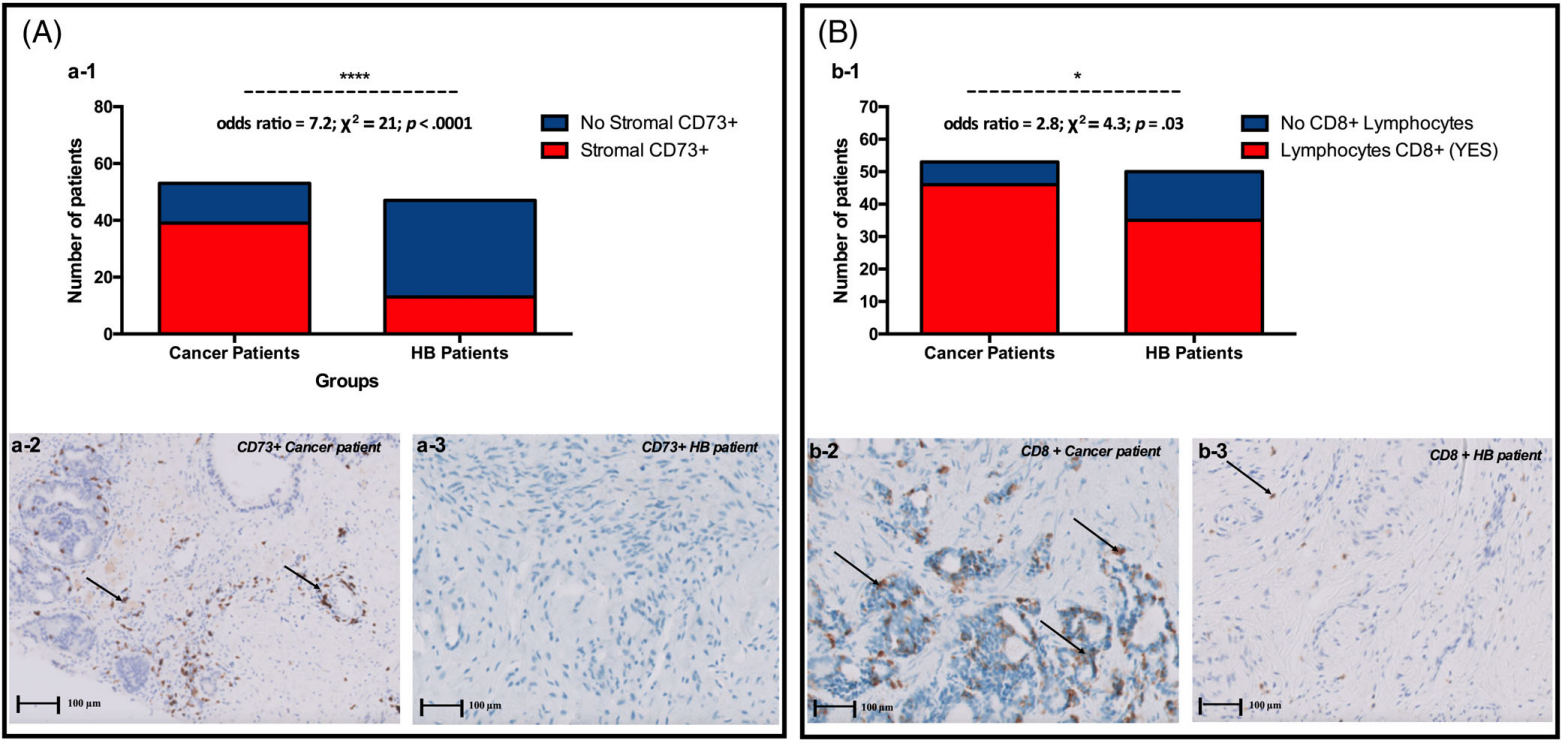
(A) Stromal CD73+ cells distribution, cancer versus BPH patients. (a1) Prostate cancer patients were significantly more likely to have stromal CD73+ cells in their than BPH patients (odds ratio = 7.2; χ^2^ = 21; *p* < .0001). (a2) CD73 expression in prostate cancer) and (a3) in benign prostatic hyperplasia. Brown staining marker of CD73 expression is very prominent in cancer, whereas it is absent in benign prostatic hypertrophy. This testifies to a strong expression of CD73 in prostate cancer. (B) T CD8+ distribution, cancer versus BPH patients. (b1) Prostate cancer patients are nearly three times more likely to have Lymphocytes T CD8+ in their stromal area than BPH patients (odds ratio = 2.8; χ^2^ = 4.3; *p* = .03). (b2) Infiltration of CD8 in prostate cancer and (b3) in benign prostatic hypertrophy (B) The marker is more accentuated in prostate cancer tissues, showing a strong infiltration of CD8 in prostate cancer.

Prostate cancer patients were significantly more likely to have stromal CD73+ cells in their than BPH patients (odds ratio = 7.2; χ^2^ = 21; *p* < .0001) (Figure 5A (a1–a3)).

#### 3.2.2 CD73+ Lymphocytes distribution, cancer versus HBP patients

No differences were seen in the presence of CD73+ Lymphocytes between cancer and BPH patients (odds ratio = 1.2; χ^2^ = 0.2; p = 0.6) (Data S1).

#### 3.2.3 T CD8+ distribution, cancer versus BPH patients

Data showed that prostate cancer patients are nearly three times more likely to have Lymphocytes T CD8+ in their stromal area than BPH patients (odds ratio = 2.8; χ^2^ = 4.3; *p* = .03) (Figure 5B (b1–b3)).

### 3.3 NK cells (CD56+) distribution in cancer patients compared with BPH patients

All patients (Cancer and BPH patients) had NK cells in the stromal area. However, some differences were observed. All BPH patients showed a high level of NK cells, whereas 69.8%, 22.6%, and 7.5% of cancer patients showed respectively high, moderate, and low levels of NK cells.

#### 3.3.1 PSA concentrations, Prostate cancer versus BPH patients

**FIGURE 6:**
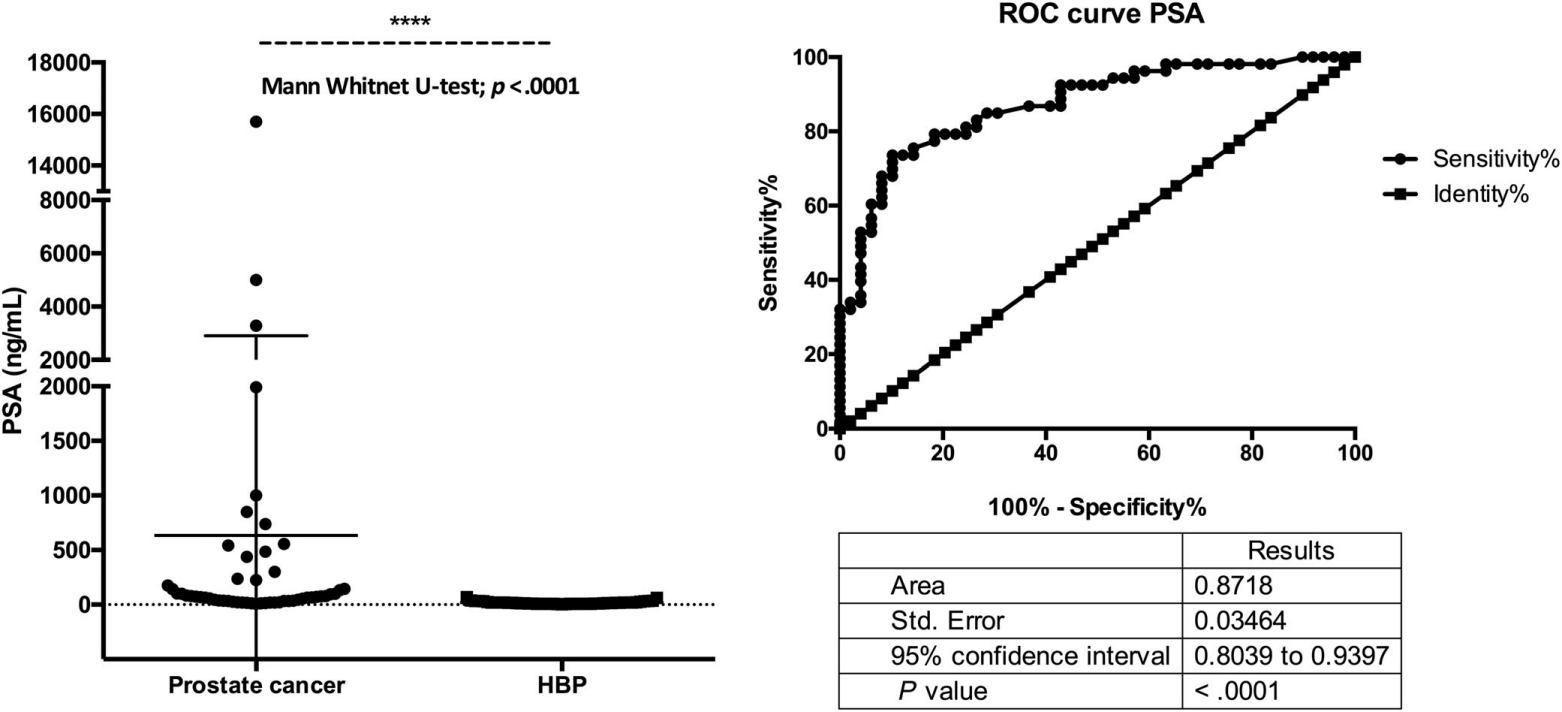
Prostate‐specific antigen (PSA). Concentrations, Prostate cancer versus BPH patients. (A) PSA was significantly higher in prostate cancer patients than in BPH patients (Mann–Whitney *U* test *p* < .001). (B) ROC curve analysis showed an area under the curve (AUC) of 0.87 (*p* < .0001).

The Concentration of PSA was significantly higher in prostate cancer patients than in BPH patients (Mann–Whitney *U* test *p* < .001) (Figure 6A). The receiver operating characteristics (ROC) curve analysis showed an area under the curve (AUC) of 0.87 (*p* < .0001) (Figure 6A,B). The cutoff analysis is shown in Table 2.

**TABLE 2:** Prostate‐specific antigen (PSA) ROC curve cutoff analysis BPH versus Prostate cancer.

PSA cutoff	Sensitivity%	95% CI	Specificity%	95% CI	Likelihood ratio
> 20.66	84.91	72% to 93%	71.43	57% to 83%	2.972
> 21.62	83.02	70% to 92%	73.47	59% to 85%	3.129
> 24.50	81.13	68% to 90%	75.51	61% to 87%	3.313
> 26.50	79.25	66% to 89%	75.51	61% to 87%	3.236
> 31.68	79.25	66% to 89%	79.59	66% to 90%	3.883
> 32.16	79.25	63% to 89%	81.63	68% to 91%	4.314
> 33.06	75.47	62% to 86%	85.71	73% to 94%	5.283
> 33.17	73.58	60% to 85%	85.71	73% to 94%	5.151
> 34.41	73.58	50% to 85%	87.76	75% to 95%	6.009
> 35.79	73.58	60% to 85%	89.80	78% to 97%	7.211
> 36.13	71.70	58% to 83%	89.80	78% to 97%	7.026
> 36.78	69.81	56% to 82%	89.80	78% to 97%	6.842
> 41.17	67.92	54% to 80%	91.84	80% to 98%	8.321

## 4 DISCUSSION

There is mounting evidence that immune surveillance evasion through immunoediting and immune dormancy is a cornerstone in cancer development and progression.^27^, ^28^, ^29^ CD73, a T‐regulatory surface enzyme, is implicated in generating extracellular adenosine. Both adenosine and CD73 are known to act as immunosuppressive protumorigenic factors.^22^, ^30^

Even though ethnicity and epigenetics factors have been shown to influence cancer physiopathological features (aggressiveness and progression),^31^, ^32^, ^33^ anti‐cancerous Immunology has never been investigated in the black African population living in Africa. Therefore, there is a need for data from this population. Moreover, at the dawn of precision medicine, an emerging care approach requires population and individual‐specific data on disease features. Thus, we investigated the tumor microenvironment of prostate cancer and benign prostatic hyperplasia in black Africans. In addition, we evaluated the expression of CD73 and the infiltrating features of immune cells, including CD8 T lymphocytes and natural killer cells.

We show for the first time in black Africans a high expression of CD73 in epithelial‐stromal cells and virtually no infiltration of CD8 T lymphocytes and natural killer cells in the tumoral area. Evidence showed that CD73 expression in the prostate epithelium suppresses immunosurveillance by CD8 T lymphocytes and NK‐cells.^22^, ^30^, ^34^ The high stromal concentration of CD73 cells, CD8+ T‐Lymphocytes, and natural killer cells and their absence in the tumoral area indicate their inability and incompetence to enter the tumor area. This would make black Africans prone to fast‐developing and highly aggressive forms of prostate cancer. Early clinical trial data suggest combining a CD73 inhibitor that limits adenosine production with a PD‐1 checkpoint inhibitor and chemotherapy can shrink pancreatic cancer.^35^ As B‐cells also mediate immune suppression via CD73.^36^, ^37^ It would have been informative to look at CD73 expression on B‐cells and their distribution in prostate cancer.

Nevertheless, the characteristics of prostate cancer in Black Africans presented here suggest the potential clinical use of CD73 expression as a target for immunotherapy in this population.^38^ Indeed, there are pieces of evidence supporting that black Africans might be more susceptible to prostate cancer than others. Published data showed that prostate inflammation, which would constitute an important induction factor of neoplastic transformation and malignant progression in the prostate, is more pronounced in black men.^39^ Also, it has been laid out that genetics, diet (alteration of the metabolic pathway), and lifestyle converge to drive the molecular pathogenesis of prostate cancer.^39^, ^40^ The changing lifestyle in developing countries characterized by increased exposure to sources of pollution (urbanization), exposure, increased usage of tobacco and alcohol, and more consumption of meat, sugar, and processed foods is considered a non‐negligible risk factor for the development of cancers in Africa.^41^

A recent study in the United States showed that prostate tumors from self‐identified Black men or men of African genetic ancestry had increased quantities of plasma cells and increased immune activity. In addition, NK activity was associated with improved outcomes after surgery.^42^ Interestingly, CD8+ T‐cell content and high activity (as measured by high cytolytic activity) were not associated with metastasis‐free survival.^42^ Also, it has been shown that a higher cytolytic score correlates with an immunosuppressive tumor microenvironment and reduced survival in brain cancer.^43^ This is to show the complexity of the tumor microenvironment. Is a humoral response supported by NK cells the appropriate antitumor‐immune response? More investigations are required to answer this question.

Data comparing cancer patients to BPH patients showed they were three times more likely to have Lymphocytes T CD8+ in their stromal area than BPH patients. Others have reported that T‐lymphocytes play a crucial role in prostate tumor development.^44^ This statement was supported by findings showing that prostatic stroma of cancer patients are characterized by an inflammatory response involving T‐lymphocytes.^44^ Also, the CD73 was seven (7) times more likely to be expressed in prostate cancer stromal tissues than in benign prostatic hyperplasia tissues. This suggests an association between prostate cancer and high CD73 expression. This could be explained by the fact that prostate cancer is a solid tumor. Several authors have shown that, generally, solid tumors are characterized by severe tumor hypoxia, consecutive to the high proliferation of cancer cells.^45^, ^46^ This hypoxia, known to regulate the expression of CD73 and the production of adenosine, leads to impaired metabolism and impaired transport of oxygen and nutrients due to abnormal tumor vascularization.^47^, ^48^

Both prostate cancer and BPH patients had an abnormally high concentration of PSA. However, the concentration of PSA was significantly higher in prostate cancer patients than in BPH. Moreover, ROC analysis showed an area under the curve of 0.87, showing that PSA may still have significant power in segregating prostate cancer from BPH. The problem of differential diagnosis based on PSA may arise for patients at an early stage of either disease.^49^

We must recognize that the present study has some limits. The first would be the absence of follow‐up, particularly for the group of patients with prostate cancer. In the absence of this follow‐up, we could not assess the prognostic value of our findings. Indeed, understanding the correlation between the observed immunological features and prostate cancer's clinical development is essential. This would have allowed us to glimpse new avenues of targeted immunotherapy research. Another limit is the non‐phenotyping of immune cells in the prostate cancer stromal area. Phenotyping would have enabled us to determine immune cells' activation, energy, and exhaustion state. In addition, a relatively small group of participants and the restricted choice of cell markers may be considered a limit. Looking at B‐lymphocytes, dendritic cells, tumor‐infiltrating macrophages, neutrophils, and other immune checkpoints, such as PDL1, CTLA4, ICOS, and VISTA, would have been more informative.

## 5 CONCLUSION

The tumor area of black sub‐Saharan African men with prostate cancer seemed to be characterized by an immune‐privileged microenvironment flanked by CD73+ cells‐rich stromal area. This is a unique investigation of the immunological microenvironment of prostate cancer in Sub‐Saharan Africans. Our study provides the first insights into the microenvironment signature of prostate cancer in Sub‐Saharan Africans. Ultimately, this study may provide important data for population‐based comparative analysis of prostate cancer microenvironments.

## AUTHOR CONTRIBUTIONS

**Pélagie Mougola Bissiengou:** Conceptualization (equal); data curation (lead); funding acquisition (equal); investigation (lead); methodology (lead); writing – original draft (equal). **Jérôme Gaston Montcho Comlan:** Data curation (equal); formal analysis (supporting); investigation (supporting); methodology (supporting); writing – review and editing (supporting). **Gabrielle Atsame Ebang:** Formal analysis (equal); investigation (supporting); methodology (supporting); validation (supporting); visualization (supporting). **Maguette Sylla Niang:** Conceptualization (equal); investigation (equal); methodology (equal); project administration (equal); supervision (equal); validation (equal); writing – review and editing (equal). **Joel Fleury Djoba Siawaya:** Conceptualization (equal); formal analysis (lead); funding acquisition (lead); investigation (equal); project administration (lead); resources (equal); supervision (equal); validation (equal); writing – original draft (equal).

## CONFLICT OF INTEREST STATEMENT

The authors declare that they do not have any competing or conflicts of interest.

## Supporting information


**Data S1:** Supporting Information

**Supplementary figures.** Distribution of Lymphocytes expressing CD73+: A: prostate cancer. B: Benign prostatic hypertrophy

## Data Availability

Data can be accessed and made available by contacting the corresponding author by e‐mail (joel.djoba@gmail.com).
