# Supplementary figures and images for: Prostate malignant tumor and benign prostatic hyperplasia microenvironments in black African men: Limited infiltration of CD8+ T lymphocytes, NK‐cells, and high frequency of CD73+ stromal cells
Source: Cancer Rep (Hoboken). 2023 Apr 24;6(Suppl 1):e1817. doi: 10.1002/cnr2.1817 (PMC10440842; doi:10.1002/cnr2.1817)

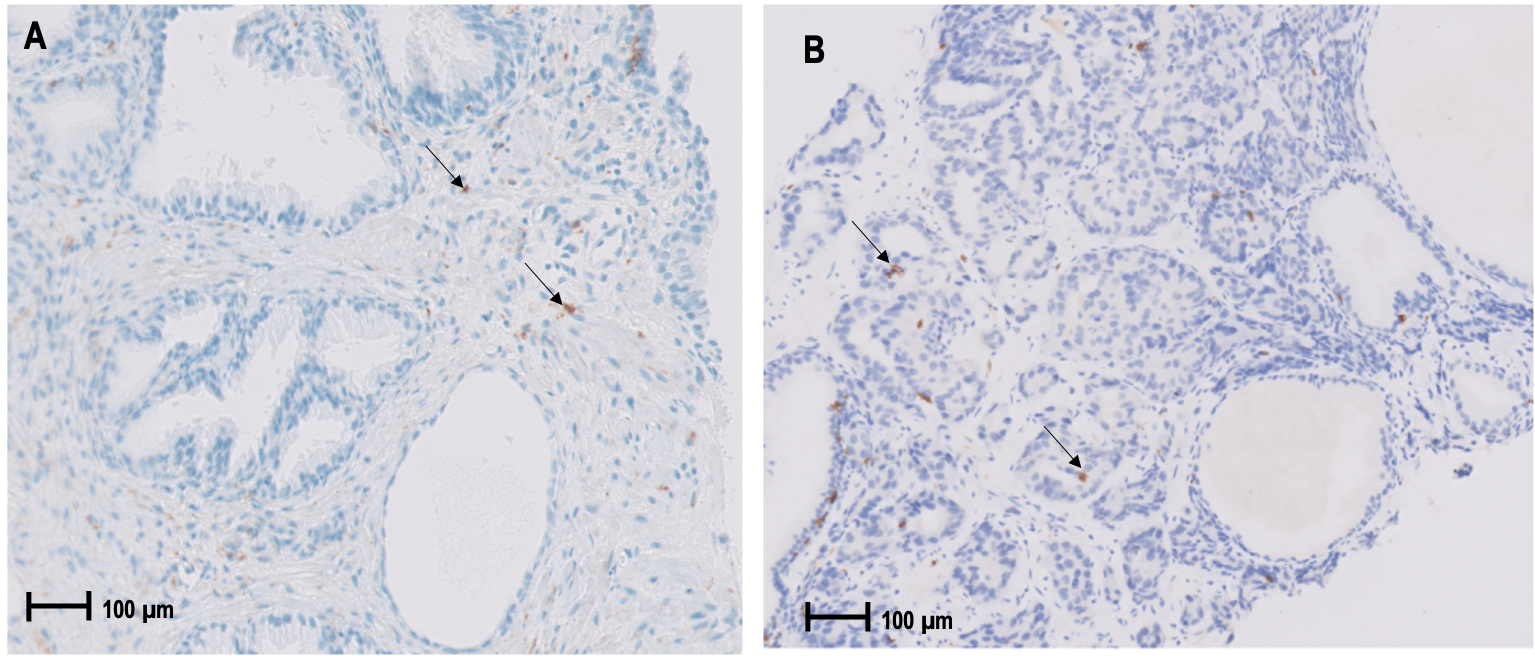

Supplement: Supplementary file 2 — Supplementary figures. Distribution of Lymphocytes expressing CD73+: A: prostate cancer. B: Benign prostatic hypertrophy [file CNR2-6-e1817-s002.png]
